# 2-Amino-4-methyl­pyridinium 4-amino­benzoate

**DOI:** 10.1107/S1600536808014839

**Published:** 2008-05-21

**Authors:** Hong Shen, Jing-Jing Nie, Duan-Jun Xu

**Affiliations:** a Department of Chemistry, Zhejiang University, People’s Republic of China

## Abstract

In the structure of the title salt, C_6_H_9_N_2_^+^·C_7_H_6_NO_2_^−^, the 4-amino­benzoate anions are linked to adjacent anions and 2-amino-4-methyl­pyridinium cations *via* N—H⋯O hydrogen bonds, forming a three-dimensional supra­molecular structure. The crystal structure also shows a weak C—H⋯O hydrogen bond between adjacent anions. Within the 4-amino­benzoate anion, the carboxyl­ate group is twisted by 14.0 (4)° with respect to the benz­ene ring.

## Related literature

For general background, see: Choudhury *et al.* (2007 ▶); Halvorson *et al.* (1987 ▶); Geiser *et al.* (1986 ▶); Geiser & Willett (1984 ▶). For related structures, see: Kaabi & Khedhiri (2004 ▶); Chtioui *et al.* (2006 ▶). For a description of the Cambridge Structural Database, see Allen (2002 ▶).
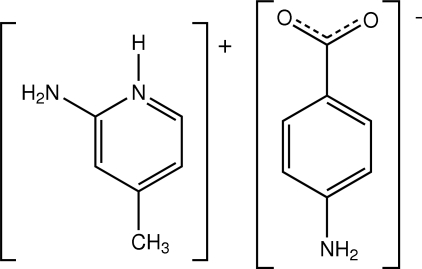


## Experimental

### 

#### Crystal data

C_6_H_9_N_2_^+^·C_7_H_6_NO_2_^−^
*M* *_r_* = 245.28
Orthorhombic, 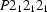

*a* = 5.5734 (14) Å
*b* = 8.8154 (16) Å
*c* = 25.374 (5) Å
*V* = 1246.6 (5) Å^3^
*Z* = 4
Mo *K*α radiation
μ = 0.09 mm^−1^
*T* = 295 (2) K
0.46 × 0.38 × 0.30 mm

#### Data collection

Rigaku R-AXIS RAPID IP diffractometer
Absorption correction: none
14099 measured reflections
1451 independent reflections
1126 reflections with *I* > 2σ(*I*)
*R*_int_ = 0.059

#### Refinement

*R*[*F* ^2^ > 2σ(*F* ^2^)] = 0.037
*wR*(*F* ^2^) = 0.095
*S* = 1.04
1451 reflections
165 parameters
H-atom parameters constrained
Δρ_max_ = 0.13 e Å^−3^
Δρ_min_ = −0.12 e Å^−3^

### 

Data collection: *PROCESS-AUTO* (Rigaku, 1998 ▶); cell refinement: *PROCESS-AUTO*; data reduction: *CrystalStructure* (Rigaku/MSC, 2002 ▶); program(s) used to solve structure: *SIR92* (Altomare *et al.*, 1993 ▶); program(s) used to refine structure: *SHELXL97* (Sheldrick, 2008 ▶); molecular graphics: *ORTEP-3 for Windows* (Farrugia, 1997 ▶); software used to prepare material for publication: *WinGX* (Farrugia, 1999 ▶).

## Supplementary Material

Crystal structure: contains datablocks I, global. DOI: 10.1107/S1600536808014839/ng2457sup1.cif

Structure factors: contains datablocks I. DOI: 10.1107/S1600536808014839/ng2457Isup2.hkl

Additional supplementary materials:  crystallographic information; 3D view; checkCIF report

## Figures and Tables

**Table 1:** Hydrogen-bond geometry (Å, °)

*D*—H⋯*A*	*D*—H	H⋯*A*	*D*⋯*A*	*D*—H⋯*A*
N1—H1*A*⋯O2^i^	0.89	2.19	3.021 (3)	157
N2—H2*N*⋯O2	0.92	1.69	2.606 (3)	174
N3—H3*A*⋯O1^ii^	0.93	1.95	2.844 (3)	160
N3—H3*B*⋯O1	0.92	1.95	2.872 (3)	174
C3—H3⋯O2^i^	0.93	2.52	3.301 (3)	142

Symmetry codes: (i) 
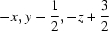
; (ii) 
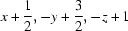
.

**Fig. 1.**
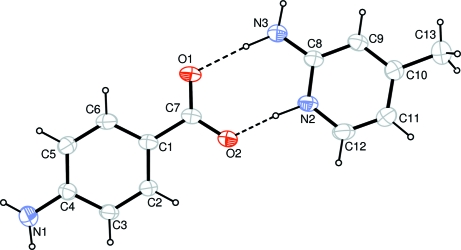
The molecular structure of the title compound with 30% probability displacement (arbitrary spheres for H atoms). Dashed lines indicate hydrogen bonding.

## supplementary crystallographic information

### Comment

The presence of the outside lone-pair electrons on the pyridine-N atom suggests that 2-amino-4-methyl-pyridine is an appropriate ligand for preparing metal complexes. However a search of the Cambridge Structure Database (November 2007 update; Allen, 2002) shows that in the most cases the 2-amino-4-methyl-pyridine presents as a counter cation but does not coordinate to the metal ion (Choudhury *et al.*, 2007; Halvorson *et al.*, 1987; Geiser *et al.*, 1986; Geiser & Willett, 1984). This implies that the 2-amino-4-methyl-pyridine, as a weak base, is easy to be protonated in acid condition. The crystal structures of two inorganic salt of 2-amino-4-methyl-pyridine, 2-amino-4-methyl-pyridinium phosphate (Kaabi & Khedhiri, 2004) and 2-amino-4-methyl-pyridinium arsenate (Chtioui *et al.*, 2006), have been reported previously. Recently we prepared the title organic salt of 2-amino-4-methyl-pyridine, and its crystal structure is reported here.

The crystal of the title compound consists of 2-amino-4-methyl-pyridinium cations and amino-benzoate anions (Fig. 1). The smaller difference in C—O bond distances of the carboxyl group (Table 1) indicates the carboxyl group is deprotonated in the crystal. Within the anion the carboxyl group is twisted with respect to the benzene ring by a dihedral angle of 14.0 (4)°. In the crystal, the aminobenzoate anions are linked with both of adjacent aminobenzoate anions and aminomethylpyridinium cations *via* N—H···O hydrogen bonding, to form the three dimensional supramolecular structure. The crystal structure also contains weak C—H···O hydrogen bonding between adjacent anions.

### Experimental

2-Amino-4-methyl-pyridine (0.054 g, 0.5 mmol) and 4-amino-benzoic acid (0.069 g, 0.5 mmol) were dissolved in ethanol (5 ml) at room temperature. The solution was filtered and light brown single crystals were obtained from the filtration after 2 weeks.

### Refinement

H atoms bonded to N atoms were located in a difference Fourier map and were refined as riding in as-found relative positions, with *U*_iso_(H) = 1.5*U*_eq_(N). Methyl H atoms were placed in calculated positions with C—H = 0.96 Å and the torsion angle was refined to fit the electron density, *U*_iso_(H) = 1.5*U*_eq_(C). Aromatic H atoms were placed in calculated positions with C—H = 0.93 Å, and refined in riding mode with *U*_iso_(H) = 1.2*U*_eq_(C). In the absence of significant anomalous scattering effects, Friedel pairs were merged.

### Crystal data

**Table:**

C_6_H_9_N_2_^+^·C_7_H_6_NO_2_^–^	*F*_000_ = 520
*M**_r_* = 245.28	*D*_x_ = 1.307 Mg m^−^^3^
Orthorhombic, *P*2_1_2_1_2_1_	Mo *K*α radiation λ = 0.71073 Å
Hall symbol: P 2ac 2ab	Cell parameters from 2654 reflections
*a* = 5.5734 (14) Å	θ = 2.0–25.2º
*b* = 8.8154 (16) Å	µ = 0.09 mm^−^^1^
*c* = 25.374 (5) Å	*T* = 295 (2) K
*V* = 1246.6 (5) Å^3^	Chunk, light brown
*Z* = 4	0.46 × 0.38 × 0.30 mm

### Data collection

**Table:**

Rigaku R-AXIS RAPID IP diffractometer	1451 independent reflections
Radiation source: fine-focus sealed tube	1126 reflections with *I* > 2σ(*I*)
Monochromator: graphite	*R*_int_ = 0.059
Detector resolution: 10.00 pixels mm^-1^	θ_max_ = 26.0º
*T* = 295(2) K	θ_min_ = 1.6º
ω scans	*h* = −6→6
Absorption correction: none	*k* = −10→10
14099 measured reflections	*l* = −30→29

### Refinement

**Table:**

Refinement on *F*^2^	Hydrogen site location: inferred from neighbouring sites
Least-squares matrix: full	H-atom parameters constrained
*R*[*F*^2^ > 2σ(*F*^2^)] = 0.037	*w* = 1/[σ^2^(*F*_o_^2^) + (0.0449*P*)^2^ + 0.1505*P*] where *P* = (*F*_o_^2^ + 2*F*_c_^2^)/3
*wR*(*F*^2^) = 0.095	(Δ/σ)_max_ = 0.001
*S* = 1.04	Δρ_max_ = 0.13 e Å^−^^3^
1451 reflections	Δρ_min_ = −0.12 e Å^−^^3^
165 parameters	Extinction correction: SHELXL97 (Sheldrick, 2008), Fc^*^=kFc[1+0.001xFc^2^λ^3^/sin(2θ)]^-1/4^
Primary atom site location: structure-invariant direct methods	Extinction coefficient: 0.015 (3)
Secondary atom site location: difference Fourier map	

### Special details

**Table:**

Geometry. All e.s.d.'s (except the e.s.d. in the dihedral angle between two l.s. planes) are estimated using the full covariance matrix. The cell e.s.d.'s are taken into account individually in the estimation of e.s.d.'s in distances, angles and torsion angles; correlations between e.s.d.'s in cell parameters are only used when they are defined by crystal symmetry. An approximate (isotropic) treatment of cell e.s.d.'s is used for estimating e.s.d.'s involving l.s. planes.
Refinement. Refinement of *F*^2^ against ALL reflections. The weighted *R*-factor *wR* and goodness of fit *S* are based on *F*^2^, conventional *R*-factors *R* are based on *F*, with *F* set to zero for negative *F*^2^. The threshold expression of *F*^2^ > σ(*F*^2^) is used only for calculating *R*-factors(gt) *etc*. and is not relevant to the choice of reflections for refinement. *R*-factors based on *F*^2^ are statistically about twice as large as those based on *F*, and *R*- factors based on ALL data will be even larger.

### Fractional atomic coordinates and isotropic or equivalent isotropic displacement parameters (Å^2^)

**Table:**

	*x*	*y*	*z*	*U*_iso_*/*U*_eq_	
N1	−0.5107 (5)	0.2323 (3)	0.74214 (9)	0.0710 (7)	
H1A	−0.4459	0.1947	0.7712	0.106*	
H1B	−0.6362	0.1981	0.7267	0.106*	
N2	0.5736 (4)	0.8834 (2)	0.61196 (7)	0.0520 (6)	
H2N	0.4548	0.8231	0.6256	0.078*	
N3	0.4308 (5)	0.8308 (3)	0.52906 (8)	0.0660 (7)	
H3A	0.4443	0.8528	0.4934	0.099*	
H3B	0.3098	0.7766	0.5454	0.099*	
O1	0.0737 (4)	0.6432 (2)	0.57815 (6)	0.0692 (6)	
O2	0.2584 (3)	0.6999 (2)	0.65310 (6)	0.0562 (5)	
C1	−0.0644 (4)	0.5269 (3)	0.65687 (9)	0.0451 (6)	
C2	−0.0117 (4)	0.4843 (3)	0.70832 (9)	0.0497 (6)	
H2	0.1249	0.5233	0.7244	0.060*	
C3	−0.1565 (5)	0.3857 (3)	0.73617 (9)	0.0523 (7)	
H3	−0.1139	0.3568	0.7702	0.063*	
C4	−0.3654 (5)	0.3295 (3)	0.71361 (9)	0.0488 (6)	
C5	−0.4210 (5)	0.3741 (3)	0.66250 (10)	0.0581 (7)	
H5	−0.5612	0.3388	0.6468	0.070*	
C6	−0.2727 (5)	0.4694 (3)	0.63474 (10)	0.0550 (7)	
H6	−0.3129	0.4960	0.6004	0.066*	
C7	0.0980 (5)	0.6303 (3)	0.62688 (9)	0.0479 (6)	
C8	0.5886 (5)	0.9037 (3)	0.55940 (9)	0.0482 (6)	
C9	0.7670 (5)	1.0014 (3)	0.53990 (10)	0.0520 (6)	
H9	0.7815	1.0156	0.5037	0.062*	
C10	0.9189 (5)	1.0757 (3)	0.57306 (10)	0.0551 (7)	
C11	0.8960 (6)	1.0501 (3)	0.62760 (11)	0.0668 (8)	
H11	0.9977	1.0989	0.6512	0.080*	
C12	0.7262 (6)	0.9547 (3)	0.64526 (10)	0.0636 (8)	
H12	0.7131	0.9372	0.6813	0.076*	
C13	1.1031 (6)	1.1849 (3)	0.55251 (12)	0.0742 (8)	
H13A	1.1123	1.1766	0.5148	0.111*	
H13B	1.2567	1.1615	0.5676	0.111*	
H13C	1.0582	1.2865	0.5619	0.111*	

### Atomic displacement parameters (Å^2^)

**Table:**

	*U*^11^	*U*^22^	*U*^33^	*U*^12^	*U*^13^	*U*^23^
N1	0.0621 (14)	0.0843 (17)	0.0664 (14)	−0.0162 (14)	−0.0039 (12)	0.0169 (13)
N2	0.0637 (14)	0.0562 (12)	0.0360 (11)	0.0004 (12)	0.0035 (11)	0.0007 (9)
N3	0.0751 (15)	0.0825 (16)	0.0403 (11)	−0.0200 (16)	0.0037 (12)	−0.0003 (11)
O1	0.0800 (14)	0.0940 (14)	0.0336 (9)	−0.0151 (13)	−0.0001 (10)	0.0088 (9)
O2	0.0660 (11)	0.0651 (10)	0.0374 (9)	−0.0093 (11)	0.0020 (10)	0.0024 (8)
C1	0.0495 (14)	0.0509 (13)	0.0350 (12)	0.0068 (13)	0.0035 (12)	0.0009 (10)
C2	0.0508 (14)	0.0590 (14)	0.0391 (13)	−0.0028 (13)	−0.0028 (11)	0.0011 (12)
C3	0.0537 (16)	0.0673 (16)	0.0360 (12)	−0.0002 (14)	−0.0013 (11)	0.0086 (13)
C4	0.0489 (15)	0.0536 (14)	0.0440 (14)	0.0021 (13)	0.0023 (12)	0.0017 (12)
C5	0.0525 (15)	0.0734 (18)	0.0484 (15)	−0.0043 (16)	−0.0046 (14)	−0.0018 (13)
C6	0.0599 (17)	0.0668 (17)	0.0383 (14)	0.0070 (16)	−0.0040 (13)	0.0035 (12)
C7	0.0559 (16)	0.0525 (14)	0.0354 (13)	0.0065 (14)	0.0036 (12)	0.0021 (11)
C8	0.0554 (15)	0.0498 (13)	0.0395 (13)	0.0042 (14)	0.0032 (12)	0.0006 (11)
C9	0.0635 (16)	0.0517 (13)	0.0409 (13)	0.0048 (15)	0.0058 (13)	0.0019 (11)
C10	0.0574 (16)	0.0479 (14)	0.0598 (16)	0.0027 (14)	0.0011 (15)	0.0004 (12)
C11	0.076 (2)	0.0692 (18)	0.0554 (17)	−0.0099 (18)	−0.0100 (16)	−0.0047 (14)
C12	0.082 (2)	0.0698 (18)	0.0387 (14)	0.0010 (19)	−0.0040 (15)	−0.0012 (13)
C13	0.0708 (19)	0.0678 (18)	0.084 (2)	−0.0106 (19)	0.0038 (18)	−0.0001 (16)

### Geometric parameters (Å, °)

**Table:**

N1—C4	1.384 (3)	C3—H3	0.9300
N1—H1A	0.8846	C4—C5	1.390 (3)
N1—H1B	0.8568	C5—C6	1.373 (4)
N2—C8	1.348 (3)	C5—H5	0.9300
N2—C12	1.354 (3)	C6—H6	0.9300
N2—H2N	0.9167	C8—C9	1.405 (4)
N3—C8	1.334 (3)	C9—C10	1.361 (4)
N3—H3A	0.9287	C9—H9	0.9300
N3—H3B	0.9252	C10—C11	1.408 (4)
O1—C7	1.249 (3)	C10—C13	1.501 (4)
O2—C7	1.272 (3)	C11—C12	1.343 (4)
C1—C6	1.385 (4)	C11—H11	0.9300
C1—C2	1.390 (3)	C12—H12	0.9300
C1—C7	1.494 (3)	C13—H13A	0.9600
C2—C3	1.381 (3)	C13—H13B	0.9600
C2—H2	0.9300	C13—H13C	0.9600
C3—C4	1.389 (3)		
			
C4—N1—H1A	115.4	C1—C6—H6	119.3
C4—N1—H1B	117.1	O1—C7—O2	123.4 (2)
H1A—N1—H1B	125.7	O1—C7—C1	119.6 (2)
C8—N2—C12	121.1 (2)	O2—C7—C1	117.0 (2)
C8—N2—H2N	119.7	N3—C8—N2	117.8 (2)
C12—N2—H2N	119.2	N3—C8—C9	124.0 (2)
C8—N3—H3A	114.1	N2—C8—C9	118.3 (2)
C8—N3—H3B	118.1	C10—C9—C8	121.1 (2)
H3A—N3—H3B	127.2	C10—C9—H9	119.4
C6—C1—C2	117.3 (2)	C8—C9—H9	119.4
C6—C1—C7	121.7 (2)	C9—C10—C11	118.3 (3)
C2—C1—C7	121.0 (2)	C9—C10—C13	121.3 (2)
C3—C2—C1	121.8 (2)	C11—C10—C13	120.4 (3)
C3—C2—H2	119.1	C12—C11—C10	119.5 (3)
C1—C2—H2	119.1	C12—C11—H11	120.3
C2—C3—C4	120.2 (2)	C10—C11—H11	120.3
C2—C3—H3	119.9	C11—C12—N2	121.7 (2)
C4—C3—H3	119.9	C11—C12—H12	119.1
N1—C4—C3	119.7 (2)	N2—C12—H12	119.1
N1—C4—C5	122.2 (2)	C10—C13—H13A	109.5
C3—C4—C5	118.1 (2)	C10—C13—H13B	109.5
C6—C5—C4	121.1 (3)	H13A—C13—H13B	109.5
C6—C5—H5	119.4	C10—C13—H13C	109.5
C4—C5—H5	119.4	H13A—C13—H13C	109.5
C5—C6—C1	121.4 (2)	H13B—C13—H13C	109.5
C5—C6—H6	119.3		

### Hydrogen-bond geometry (Å, °)

**Table:**

*D*—H···*A*	*D*—H	H···*A*	*D*···*A*	*D*—H···*A*
N1—H1A···O2^i^	0.89	2.19	3.021 (3)	157
N2—H2N···O2	0.92	1.69	2.606 (3)	174
N3—H3A···O1^ii^	0.93	1.95	2.844 (3)	160
N3—H3B···O1	0.92	1.95	2.872 (3)	174
C3—H3···O2^i^	0.93	2.52	3.301 (3)	142

Symmetry codes: (i) −*x*, *y*−1/2, −*z*+3/2; (ii) *x*+1/2, −*y*+3/2, −*z*+1.
